# Mainzelliste: Ten years of pseudonymization, record linkage, and informed consent management

**DOI:** 10.1016/j.patter.2025.101432

**Published:** 2025-12-16

**Authors:** Galina Tremper, Torben Brenner, Moanes Ben Amor, Tobias Kussel, Martin Lablans

**Affiliations:** 1 Federated Information Systems, German Cancer Research Center (DKFZ), Heidelberg, Germany; 2 Complex Medical Informatics, Mannheim Institute for Intelligent Systems in Medicine (MIISM), Medical Faculty Mannheim of Heidelberg University, Mannheim, Germany; 3 German Cancer Consortium (DKTK), DKFZ, Core Center Heidelberg, Heidelberg, Germany; 4 DKFZ Hector Cancer Institute at the University Medical Center Mannheim, Mannheim, Germany; 5 Helmholtz Institute for Translational Oncology Mainz (HI-TRON Mainz) – A Helmholtz Institute of the DKFZ, Mainz, Germany

**Keywords:** pseudonymization, record linkage, informed consent management

## Abstract

Record linkage and pseudonymization are crucial tasks in collaborative biomedical research. Data for a patient are rarely stored in one place and therefore often need to be linked and integrated across multiple institutions. Mainzelliste is an open-source software solution designed to solve these challenges by providing a comprehensive and flexible toolkit for pseudonymization, record linkage, and consent management. It supports a variety of pseudonyms, record linkage methods, and modular, informed patient consents. A highly flexible REST application programming interface (API) allows tight integration into existing applications and workflows. Since its initial release in 2015, Mainzelliste has evolved into a vibrant open-source software solution “by researchers, for researchers” including a user-friendly graphical interface, support for HL7 FHIR for consent and patient data, and record linkage based on secure multi-party computation, thereby supporting secure and efficient biomedical research.

## Highlights

• Software for pseudonymization, error-tolerant record linkage, and consent management
• Flexible REST API for seamless integration with research software and workflows
• 100% open-source software with regular contributions from multiple institutions
• Widely adopted in international registries, biobanks, projects, and research networks

## Teaser

Mainzelliste is open-source software for pseudonymization, record linkage, and informed consent management. It is developed and widely used within the medical informatics community, biobanks, patient registries, and research networks. It can be used as a standalone application or integrated into existing environments and processes via a flexible REST API.

## Introduction

Access to personal patient data is a requirement for many projects in medical research. Especially with regard to rare diseases or personalized treatments, even large hospitals might not have enough cases to support complex research questions. Additionally, patients may be treated in multiple institutions, requiring their data to be linked across multiple databases.^1^^,^^2^ However, using patient data across multiple institutions faces some well-known challenges.

(1) Patient data are highly sensitive. From a data protection perspective, patient data can be divided into two groups: personally identifiable information (PII), such as name or date of birth, and medical data (MDAT), which includes clinical records, such as diagnoses or therapy details. PII is used to uniquely identify a person but is seldom required for data analysis. PII needs particular protection to prevent misuse, such as unauthorized re-identification, which could result in disadvantages for the individual (e.g., discrimination due to socially stigmatized diagnoses^3^).
(2) Distribution of patient data creates barriers. The medical records of interest, which may include, e.g., clinical records, imaging, and genomic data, are rarely consolidated in a single repository. Instead, they are often dispersed across different, legally independent institutions and their heterogeneous and often non-interoperable information technology (IT) systems, making record linkage a prerequisite for distributed data integration.^4^
(3) Using patient data requires a legal basis. In the European Union, the storage and processing of personal data are regulated, *inter alia*, by the General Data Protection Regulation (GDPR), which usually requires a written, informed patient consent as a legal basis for the data usage.^5^ Each patient’s informed consent needs to be recorded and managed through all processing steps, including versioning and potential withdrawal of the consent itself.

In this work, we present Mainzelliste, an open-source software solution for pseudonymization, record linkage, and consent management. It can be used either as a standalone software or using a flexible application programming interface (REST API^6^), integrated into existing software and workflows. Its main features are as follows.

(1) Pseudonymization. To avoid storing PII together with MDAT, Mainzelliste implements multiple pseudonym generators to serve as replacements for PII in the MDAT storage. Pseudonyms are “non-speaking” labels, i.e., they cannot be used to deduce any personal patient data. Additionally, Mainzelliste can function as the primary pseudonym management tool, storing and correlating multiple pseudonyms and optionally PII for the patients.
(2) Record linkage. Mainzelliste allows users to link datasets among institutions even in the absence of existing unique patient identifiers, with error-tolerant record linkage methods based on their potentially incomplete, erroneous, or even conflicting PII. Depending on the use cases and data protection restrictions, different methods are supported: from “simple” comparison of the clear-text PII fields to privacy-preserving record linkage (PPRL) implementations using Bloom filters or even cryptographic state-of-the-art protocols for secure record linkage based on secure multi-party computation (SMPC) techniques,^7^ meeting even the highest security requirements.
(3) Informed consent management. Mainzelliste implements lean management of modular informed consents and stores the consent information together with the patient data to allow easy checking of the legal basis for data processing. This functionality is based on the widely used FHIR standard^8^ developed by HL7 for representing clinical data models and interfaces as well as for exchanging the data between systems.

**Figure 1:**
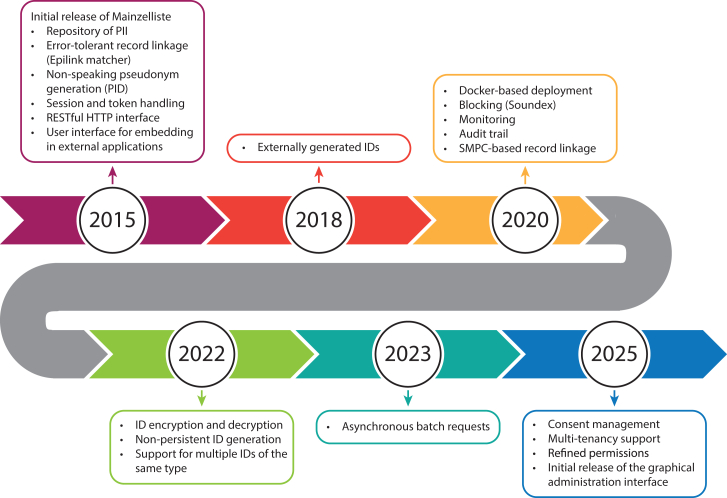
Timeline of the Mainzelliste feature development

The initial release of Mainzelliste, consisting of an interface for communication between the systems in internet in form of a REST API^9^ (representational state transfer API) and basic web forms for entering/editing patients, took place in 2015. Since then, it has been widely used in the implementation of pseudonymization processes for medical research networks,^10^^,^^11^^,^^12^ patient registries,^13^^,^^14^^,^^15^^,^^16^^,^^17^ and biobanks^18^ and has been continuously improved. In this work, we present the current, significantly extended state of Mainzelliste, including new features, such as refined access and role-based permissions, consent management, and a new user-friendly graphical administration interface. Figure 1 provides an overview of Mainzelliste’s feature development over the last decade.

## Methods

For a better understanding of the methodology and functionality of Mainzelliste, we first introduce our simplified mental model of how a patient can be described. This directly informs Mainzelliste’s data model and usage.

**Figure 2:**
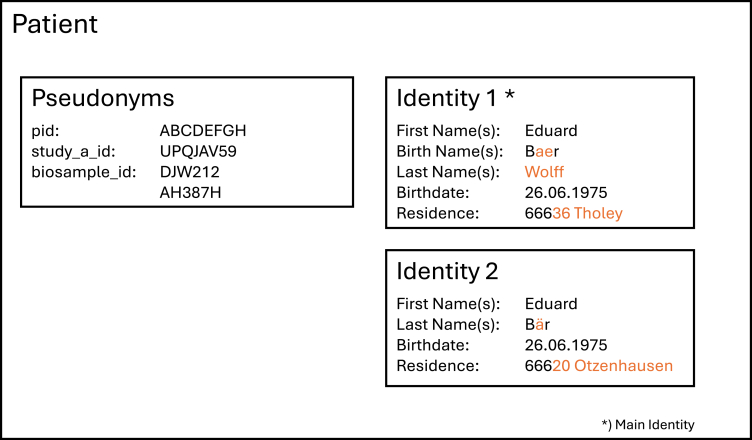
Simplified mental model of a patient who has recently married and relocated to another city and is thus stored with two identities Differences are marked in orange. The concept of multiple identities is detailed in record linkage.

As exemplified in Figure 2, Mainzelliste references a patient using their pseudonyms and/or identities. Each patient is a real person with a set of PII. As we want to track a patient across time, it might also happen that some of the identifying data change between patient visits (e.g., due to marriage, change of residence, or simple typos during data capture). In this case, simply modifying the stored PII could lead to difficulties, as existing links might break.^19^ Hence, all past identities are also kept for a patient and considered in record linkage.

### Types of pseudonyms

To mitigate the effects of potential data breaches, it is good practice to store PII separately from medical records.^20^^,^^21^ A pseudonym is created to link both data parts, which does not reveal any additional information concerning the patient’s identity (a so-called non-speaking pseudonym). However, authorized users can use the pseudonym to resolve the PII, e.g., for re-contacting the patient in the case of significant findings or to support treatment processes.^4^^,^^22^^,^^23^^,^^24^ The pseudonym, rather than the PII, is then used to link all data of a patient across different storage locations and sites.

Mainzelliste supports multiple methods of pseudonym generation that can be described along three axes.

(1) Internally vs. externally generated pseudonyms. Mainzelliste supports users in either generating pseudonyms themselves (internally) or in utilizing pseudonyms already generated by other applications (externally), for example, by electronic data capture (EDC) systems, which are used for collecting, managing, and storing patient data, or by registries, biobanks, or hospital information systems. While internally generated pseudonyms are created only once for a given patient and are immutable, external pseudonyms are not generated but are entered either manually or via the API and can be edited later to accurately reflect any changes in the source system.
(2) Persistent vs. non-persistent pseudonyms. Pseudonyms are either stored in the database (persistent) or generated upon each access in memory using a deterministic algorithm (non-persistent). To this end, Mainzelliste provides pseudonyms of type CryptoID that are derived from a primary pseudonym with symmetric encryption, obviating the need for persistence. It can be either an internally or externally generated pseudonym. As the symmetric encryption algorithm uses the same secret key for encryption and decryption, it is always possible to generate a CryptoID from its base pseudonym (or vice versa).
(3) Single vs. multiple pseudonyms. Mainzelliste supports assigning different pseudonyms to a single patient, based on the context of the request. In most cases, it is enough to add one pseudonym per context to the patient (e.g., per study; see study_a_id in Figure 2). In other cases, it might be necessary to add multiple pseudonyms of the same type (e.g., biosample_id in Figure 2).

The pseudonym string itself (meaning, the string of characters making up the ID) is also an important consideration. For example, despite their advantages, non-persistent cryptographically generated pseudonyms are usually too long to be handled by humans and are thus recommended for programmatic use only. Conversely, Mainzelliste generates user-friendly persistent pseudonyms based on the algorithm by Faldum and Pommerening,^25^ resulting in short, human-readable, verifiable, and even self-correcting alphanumeric pseudonyms.

Alternatively, pseudonyms can be highly customized to project requirements, e.g., by basing them on pre-defined vocabularies (ElasticID) or by designing them to be short enough to fit on the label of a biosample tube.

### Record linkage

**Figure 3:**
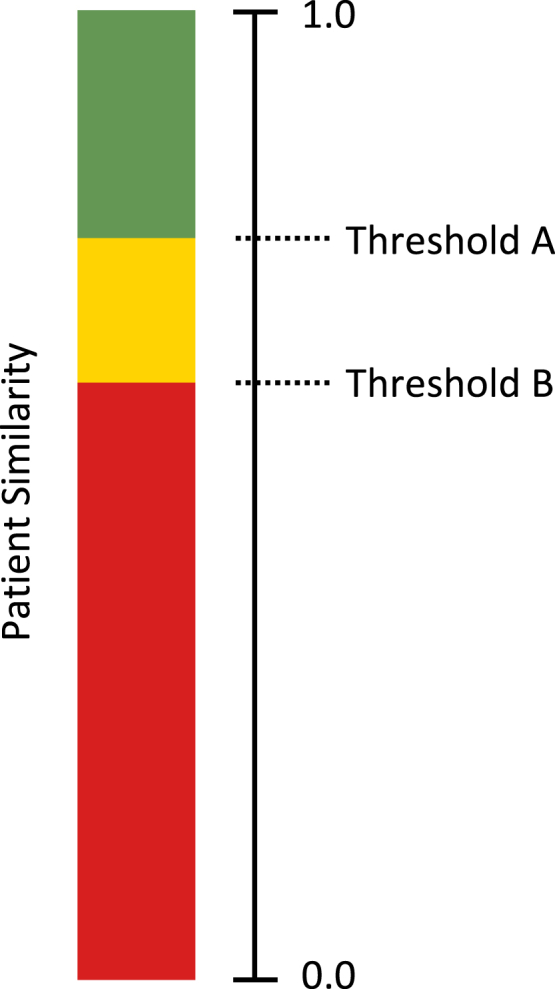
Match and non-match thresholds for patient similarity

Record linkage describes the process of linking data records belonging to the same person from different data sources or time points. In the simplest case, an existing unique patient identifier, such as a health insurance number, can serve as the linking pseudonym, making record linkage trivial. In cases where such an identifier does not exist or must not be used for record linkage purposes (for example, the usage of the German unique and immutable health insurance number is purpose bound to medical care and not allowed for research purposes^26^), Mainzelliste performs the record linkage by comparing the personal data fields, such as first/last name(s), date of birth, and address. To compare personal data fields, we use the EpiLink algorithm,^27^ which computes a patient similarity score between zero and one (Figure 3). Two definable thresholds (thresholds A and B) then classify the pairing to be a match (green), a tentative match (yellow), or a non-match (red). This allows for an error-tolerant record linkage, e.g., in the case of data entry errors, and is highly tunable based on statistical properties of the underlying dataset.

For cases that are considered as a tentative match, a conflict resolution procedure decides whether the two sets of PII marked as tentative matches (yellow area) belong to the same patient or not. This decision is usually done manually by an authorized person with permission to see the PII for both patients, e.g., a trustee or the treating physician. For this purpose, the tentatively matching patients are created as separate records in the patient list but are marked as a tentative match. The pairs of tentative matches can be listed later for conflict resolution.

A conflict is resolved by either merging or splitting the patient records. To avoid the repeated occurrence of conflicts when entering the same patient, e.g., for separate visits, Mainzelliste allows storing multiple alias identities (e.g., identity 2 in Figure 2). Record linkage and lookups are also performed on the alias identities, but for the sake of usability, only the main identity (e.g., identity 1 in Figure 2, marked with an asterisk) is visible to the users. Currently, this feature is not yet included in release versions but is being tested as part of the early adopter process (cf. safeguarding PII), in particular to investigate potential conflicts with Mainzelliste’s multi-tenancy feature (cf. using Mainzelliste in complex environments).

Alternatively, depending on the use case, Mainzelliste can automatically reject tentative matches, and the data provider needs to double-check the data and repeat the pseudonymization request.

#### PPRL

If the record linkage is done locally or if the data protection concept (DPC) covers it, the record linkage can be done by comparing the PII in clear-text form. Otherwise, the sensitive data need to be protected, for example, by hashing, encryption, or encoding them in a specifically constructed Bloom filter form,^28^ to mitigate certain attacks that could lead to unauthorized re-identification (PPRL). The Bloom-filter-based PPRL allows the comparison of the encoded patient data to calculate a patient similarity, just as in the clear-text algorithm, without revealing the patient data.

Although computationally difficult, “decoding” a Bloom filter to reconstruct (parts of) the PII is far from impossible. Over the years, new attack paths and mitigation strategies have been developed,^29^^,^^30^^,^^31^ leading to a “cat-and-mouse game” between potential attackers and software implementers. Thus, for some use cases, e.g., those involving particularly vulnerable groups, the privacy guarantees given by using Bloom filters are not sufficient for an appropriate level of data protection. For these cases, Mainzelliste offers SMPC algorithms, based on cryptographic protocols, to achieve qualitatively distinct, mathematically provable security and privacy guarantees.^7^ While on the bleeding edge of security research and maybe even to be considered a bit experimental, the feasibility of SMPC-based PPRL with Mainzelliste has been demonstrated in a pilot study involving eight German university medical centers^32^ (cf. discussion).

#### Blocking

During unoptimized record linkage, each patient needs to be compared with every other patient, leading to a quadratic algorithmic complexity. For larger datasets, this quickly leads to infeasible run times. To increase the performance of record linkage, Mainzelliste implements blocking algorithms.^33^ For blocking, the fields are preprocessed, and the patients are clustered based on an approximate similarity. Now, only the patients within one “bucket” need to be compared. For clear-text matching, phonetic blocking based on the Soundex algorithm,^34^ which aims to encode homophones with the same textual representation, was implemented, and for Bloom filter fields, locality-sensitive hashing (LSH)^35^ was used.

### Consent management

The legal basis for storing and processing MDAT for research is often an informed patient consent. Many consents are built in a modular fashion, allowing patients to opt in or opt out of various ways of data processing. For example, a patient might consent to their data being shared with third parties in pseudonymous form but ask not to be contacted again to be informed of additional research findings.

Mainzelliste provides tool-assisted handling of such modular, informed patient consents. It supports automated consent checks that integrate with workflows such as data sharing with third parties, de-pseudonymization, or the scientific use of health insurance data. Consents can be viewed, processed, tracked, and revoked at any time, thereby ensuring compliance throughout the research life cycle.

#### HL7 FHIR-compliant API

Mainzelliste follows the international HL7 FHIR standard (*consent* resource^36^) to digitally represent each patient’s consent and its directives, allowing systems, such as EDC systems, to create, examine, or delete consents (in the case of a withdrawal). To process scanned paper documents, Mainzelliste implements the German Medical Informatics Initiative’s FHIR profiles *DocumentReference*^37^ and *Provenance*^38^: the scanned documents can be embedded in Base64-encoded form within an FHIR *DocumentReference* resource. A corresponding *Provenance* resource is used to link both digital consent (FHIR *consent* resource) and scanned consent (FHIR *DocumentReference* resource).

#### Reusable consent templates

Consent templates can be used to reuse consent forms and their digitally modeled structure, completely or in parts, across studies. By building on widely used consent “building blocks,” e.g., the German nationally harmonized Broad Consent^39^ and its study-specific additions,^40^ new studies can be set up faster and in a harmonized way. The templates connect the consent’s textual representation (i.e., the actual form that patients read and sign) to the digital representation, connecting each checkbox in the form to corresponding data usage directives in the digital representation.

### Interfaces and integration

Implementing the separation of PII and MDAT in a single system or, on a larger scale, in a medical research network involves a trade-off between data protection laws and the usability of the systems. Due to the fact that each system involved should only process the data it is concerned about, it is necessary to navigate the users from one system to another. To make the switches as seamless as possible and thus not negatively affect the user experience, Mainzelliste allows for extremely versatile integration into other software and processes. The user usually interacts “transparently” with Mainzelliste by being transferred from other systems when performing a task involving patient identity management, e.g., creating a new patient, in a similar way to modern payment processors or single-sign-on systems. In the following, we give insights into the practical usage of Mainzelliste by describing a typical use case.

#### Integration with a single service

**Figure 4:**
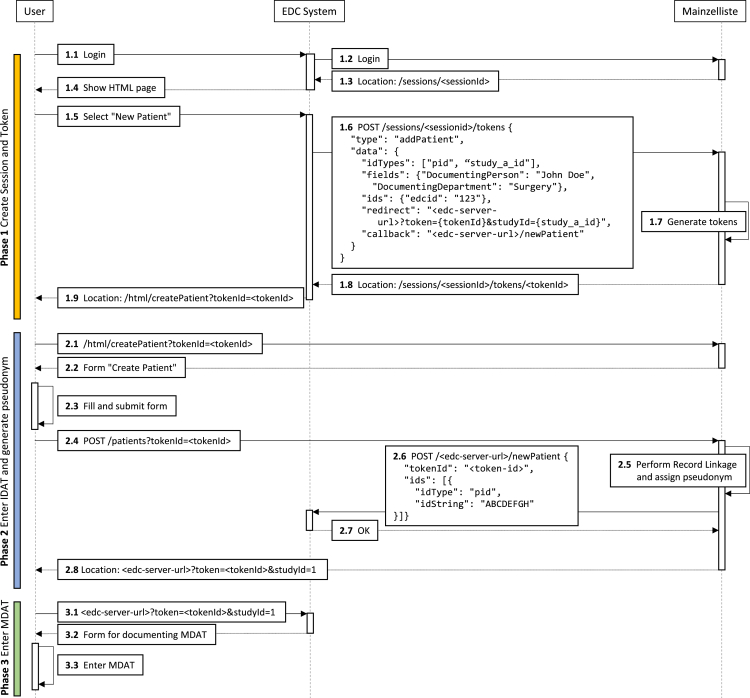
Exemplary workflow of an EDC system delegating pseudonymization to Mainzelliste

Figure 4 shows an exemplary process in which a user wants to register a patient for a study using an EDC system. In this example, the EDC system uses Mainzelliste REST API to create a session and an *addPatient* token, which is then seamlessly redeemed by the user’s web browser without further interaction with the EDC system. By employing direct communication among the parties, the MDAT server, that is, the EDC system in this example, does not learn the PII, nor does the user’s web browser learn the generated pseudonym. Similarly, it is not necessary to implement a user or rights management into Mainzelliste.

The workflow starts by creating a session for one client (or user) and creating the tokens for each operation the user would like to perform. The type of the token maps to the permitted operation (e.g., *readPatient* for retrieving the patient information). Therefore, sessions and tokens are responsible for the role management and authorization, and all subsequent requests need to redeem the issued tokens. Tokens are always validated before processing the request, so users are only able to perform allowed operations and only see the information necessary for the task.

In our exemplary use case in Figure 4, the user logs in to the EDC system (step 1.1), which, in the background, creates a new session with Mainzelliste. This interaction is hidden from the user (steps 1.2 and 1.3). To grant the user permission to add a new patient, the EDC system then creates an *addPatient* token (steps 1.6–1.8). This token is stored in Mainzelliste for the duration of the operation and enables the user to later interact with the API endpoint for creating a new patient. The EDC system itself uses an API-key-based authentication with Mainzelliste to prevent the malicious creation of tokens. During the token creation, the EDC system can also transmit supplementary information to Mainzelliste. In the example, the EDC system informs Mainzelliste about the department and the name of the user. It is also possible to transmit external pseudonyms this way (step 1.6).

In the general case, the EDC system can now redirect the user to Mainzelliste’s HTML API, which generates simple HTML pages for the user to interact with (e.g., a form for entering the PII). When the user submits such a form, Mainzelliste performs record linkage and pseudonymization. In the example, the user is not supposed to see the generated pseudonym used for interactions between the EDC system and Mainzelliste. Hence, Mainzelliste must call the EDC system directly. To represent such a call, without incurring additional implementation work for each attached service, Mainzelliste allows the definition of a callback during the token creation, stating the URL to which the result should be posted after successfully finishing the operation. In our example, the user is allowed to see the pseudonym studyId, while the second pseudonym pid is hidden from them. To implement this, the EDC system defines a callback for Mainzelliste (step 1.6), which will cause the transmission of the pid after the pseudonym is assigned (step 2.6). Instead of showing the created pseudonym directly to the user, Mainzelliste then redirects them to a specified resource in the EDC system (steps 2.8 and 3.1).

#### Using Mainzelliste in complex environments

In larger-scale deployments, it is useful to operate a single Mainzelliste for multiple projects and applications (e.g., different instances of EDC systems rolled out in a university hospital). In this case, sharing one database of PII and pseudonyms among different projects allows for more efficient usage of space, facilitates data management, and contributes to the interoperability among projects, since the record linkage across domains (if permitted) is technically easier to perform within one list. Despite storage within the same Mainzelliste instance, each user can see only their own patients from a domain and from the user’s perspective; there is no difference between a dedicated, domain-specific patient list and one central patient list shared across domains. This functionality is covered by the multi-tenancy feature of Mainzelliste.

In Mainzelliste, the relation between a study (or studies) and a patient is expressed using a special project pseudonym that is generated during the enrollment of a patient into a project. In our example, the patient is only enrolled in study “A,” and this study is represented in Mainzelliste as study_a_id. With multi-tenancy, the connection of a patient to a study will cause the specific study pseudonym to be generated automatically during the user’s createPatient requests.

Having assigned the patients to a study, or more generally to a tenant, Mainzelliste’s granular permissions, which are defined per instance in the configuration, ensure appropriate access rights. Defining granular permissions makes it possible to restrict the allowed operations for a certain pseudonym (e.g., study_a_id) or field name. Consent management is fully integrated into the permission system. This allows the definition of specific user roles, e.g., study nurses, physicians, and administrators.

For distributed pseudonymization architectures involving a trusted third party (TTP), the secure transmission of pseudonyms is crucial.^4^ For that, Mainzelliste provides asymmetric encryption methods that require two separate but related keys: a public key for encryption and a private key for decryption. The public key can be derived from the private key, but not vice versa. The site encrypts the payload with the TTP’s public key, allowing the TTP to decrypt the pseudonym using its private key. The implemented hybrid encryption combines both approaches: the (potentially large) pseudonym is encrypted using performant symmetric key encryption, while the (symmetric) secret key is encrypted using asymmetric encryption.

For more complex pseudonymization workflows, which are not covered solely by the pseudonymization tool, e.g., ramifications in the pseudonymization workflow depending on conditions or filtering the results, the *MagicPL* pseudonymization workflow description language^41^ can be used in combination with Mainzelliste. It helps to avoid hard-coding special requirements, allowing easy and flexible adjustments of the workflow.

#### Administrative usage of Mainzelliste (GUI)

**Figure 5:**
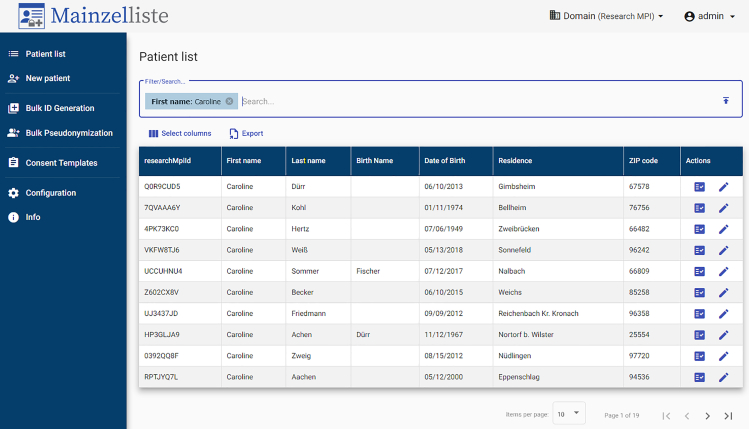
Patient list view in the administrative Mainzelliste GUI

The Mainzelliste graphical user interface (GUI), which is a separate frontend application on top of Mainzelliste’s REST API, provides a visual interface for administrative purposes. It provides basic operations, such as pseudonymizing a new patient, modifying identifying fields, generating additional IDs, deleting existing patients, and storing or withdrawing consents. The main page (Figure 5) provides a list of all stored patient data and offers real-time filtering based on PII fields and pseudonyms, making it easy to locate a specific patient or a set of patients. Furthermore, it allows importing comma-separated value (CSV) files for large-scale bulk operations, such as pseudonymization or generating secondary pseudonyms. Based on the pre-configured user roles, the Mainzelliste GUI respects the user permissions across all tenants.

#### Performance considerations

In principle, record linkage requires mutual comparison of all patient datasets and thus suffers from a quadratic algorithmic complexity. To avoid runtime performance issues with larger datasets, Mainzelliste employs three optimizations: the use of Soundex and LSH-based blocking drastically reduces the required number of comparisons and an asynchronous batch API allows the uploading of large numbers of patients with a roughly 7–8 times speedup compared to sequentially adding new patients on an Intel Xeon Platinum 8276 CPU with 4 cores at 2.20 GHz and 32 GB of RAM. The upload of one million patients into an empty Mainzelliste using the batch API takes 51 min, including local record linkage, and 15.5 min with record linkage disabled. Detailed benchmarks are available in the source repository.^42^

#### Safeguarding PII

Health-related PII ranks at the top of the lists of data sensitivity, e.g., in the GDPR^43^ and the Health Insurance Portability and Accountability Act (HIPAA).^44^ Hence, security is paramount in any software processing it, including Mainzelliste. Mainzelliste is an academically driven development that follows the best practices of the Open Worldwide Application Security Project’s Software Assurance Maturity Model (OWASP SAMM).^45^ Some examples for controls and procedures, based on the OWASP Top 10 vulnerabilities,^46^ are described along the phases of the software development life cycle.^47^

In the design phase, the principle of defense in depth^48^ is implemented through multiple, independent layers of security controls. Should one of the control layers fail, the next layers will take over to mitigate attack vectors. Two of OWASP’s Top 10 vulnerabilities (including the topmost one) deal with broken access control (authorization) and insecure authentication. Hence, Mainzelliste employs a multi-layered approach.

Firstly, users and applications authenticate either with a pre-shared API key or, to allow integration into more complex authentication scenarios, with an OAuth access token. Secondly, Mainzelliste is usually deployed within network zones protected by firewalls and reverse proxies that inspect incoming traffic, restrict access to trusted networks, and/or impose additional client restrictions, such as an additional layer of authentication.

After authentication, a multi-tenant role-based access control (RBAC) with fine-grained permissions implements the principle of least privilege and separation of duties.^49^ Each user or client is assigned a specific role that gives only the minimum privileges required to perform their tasks. To perform any operation, the user’s web browser must obtain a specific Mainzelliste token. Each token grants specific permissions to a role on a specific resource and operation. For example, the *addPatient* token (cf. integration with a single service) allows the authorized user to have write access to add exactly one patient’s identifying data to the study. Multi-tenancy ensures the secure separation of data across different domains, preventing data leakage and cross-domain attacks.

In addition to the usual application logging, the optional audit trail functionality extends Mainzelliste tokens so that a reason for requesting a token must be supplied and is logged. This allows secure auditing of all data accesses and embedding into change management workflows.

In the development phase, secure coding practices are used to reduce the risk of introducing vulnerabilities. Mainzelliste is developed in a transparent open-source workflow following the “security-in-the-open” principle.^49^ Since we decided against an internal development git repository, every single contributed line of code is instantly visible worldwide. By allowing and encouraging all contributors and users of Mainzelliste to examine the code and thoroughly review all code contributions (“pull requests”), the security posture of the software is improved.^50^

Static code analysis tools, such as SonarQube^51^ and Snyk,^52^ analyze the source code during the development phase and report known vulnerabilities. The importance of the detection and mitigation of so-called supply chain attacks, the inclusion of malicious code through code dependencies, was impressively demonstrated by the “xz/liblzma” attack in 2024.^53^ As a mitigation, Mainzelliste is automatically scanned to report all third-party dependencies’ known vulnerabilities. Critical vulnerabilities are rapidly resolved with hotfixes and patch releases without waiting for regular release cycles.

In the build-and-release phase, a continuous integration/continuous delivery (CI/CD)^54^ pipeline automatically generates reproducible builds for all code changes and triggers automated unit and integration testing. Having a public CI/CD pipeline that also follows the security-in-the-open principle is important to prevent tampering with released software binaries and to ensure a quick distribution of security patches. The generated Docker images are also automatically scanned for security vulnerabilities.^55^

Mainzelliste’s development is driven by new requirements from the projects employing it. Hence, it follows a feature-based release model where the new features are developed in a feature branch, tested within the contributing projects and by early adopters, and finally, stable versions are released as soon as a set of features has undergone sufficient testing. For capacity reasons, as academic developers, we cannot maintain multiple versions or offer long-term support (LTS) versions. Instead, only the latest Mainzelliste version is supported, and all users are asked to update immediately. In practice, this is not a limitation for users, as even the most recent Mainzelliste version is backward compatible with the very first release in 2015.

In the deployment and maintenance phase, while secure coding practices and automated scanning are significant parts of good security posture, the deployment environment and configuration are an equally important part of the overall security concept. As the deployment environment is largely outside of the control of the developers, we describe some examples of measures and controls in existing projects and infrastructures employing Mainzelliste. As it contains highly sensitive patient data, Mainzelliste’s operation is often described in a project-specific DPC,^13^^,^^14^^,^^56^^,^^57^ outlining data flows and accesses, user roles, and other details relevant for regulatory compliance. It names responsible parties, bodies overseeing data protection, as well as technical and organizational measures, ensuring the required high level of security. In many countries, procedures have evolved around the creation and publishing of DPCs, e.g., in Germany, the TMF e.V. Working Group on Data Protection offers guidance and even blueprints for DPCs in the sector of biomedical research.^58^

On the technical side, it is recommended that Mainzelliste be operated in a secure network zone with either controlled internet access or no internet access at all. If internet access is required, reverse proxies can be deployed, e.g., to restrict the access of administrative REST endpoints to specific IP ranges and networks. Lastly, many projects test the overall security by performing security audits or penetration tests that take into account the specifics of the IT environment a Mainzelliste is operated in and its configuration. A survey of seven projects and registries employing Mainzelliste showed that most projects perform security audits and penetration tests of their sensitive infrastructure, of which Mainzelliste is considered an integral component. However, these measures target the respective research network as a whole rather than the Mainzelliste software specifically. To fill that gap, Mainzelliste underwent an independent white-box penetration test in 2025 as part of the LeMeDaRT^59^ project, with the findings addressed in the following version. In this white-box test, the tester received detailed information about the system, user accounts, configuration, and the software’s source code.

## Discussion

Over the last decade, Mainzelliste has evolved into a flexible solution for pseudonymization, record linkage, and consent management, actively powering very different kinds of projects, research networks, and infrastructures with different needs (examples are listed in the introduction). In smaller applications, Mainzelliste functions merely as a simple one-off pseudonym generator. In contrast, large research networks that require multiple pseudonym types, user-friendly integration into EDC systems or biobanking solutions, and custom validators use the software in multicentric research collaborations.

Given the importance of patient identity management in the field of biomedical research, it is not surprising that multiple software solutions exist in this space, such as EUPID,^60^ the Greifswald MOSAIC Suite (E-PIX, gPAS, and gICS),^61^^,^^62^^,^^63^ LSHDB,^64^ OpenPseudonymizer,^65^ and SPIDER.^66^ For a survey and a detailed comparison, please see Abu Attieh et al.^67^ for pseudonymization services and Gkoulalas-Divanis et al.^68^ for PPRL techniques and software implementations.

What distinguishes Mainzelliste from the related solutions is its versatile integration into the software and processes in medical research networks, which has been at the core of Mainzelliste’s architecture and an intended use from the beginning.^9^ Using Mainzelliste’s API, both academic users and commercial software vendors have integrated Mainzelliste into applications widely used in biomedical research, including REDCap, the STARKIT EDC system, the OSSE registry system, the CentraXX biobanking solution, or the tripletrax EDC system.^69^^,^^70^^,^^71^^,^^72^^,^^73^ Due to Mainzelliste’s API capabilities, it can be integrated seamlessly into other “host” applications.^74^ It also offers all of the interrelated tasks of pseudonymization, record linkage, and consent management in one fully integrated software solution.

Also, Mainzelliste is, to our knowledge, the only record linkage solution implementing a full SMPC-based probabilistic record linkage, via its extension called Mainzelliste Secure EpiLinker (MainSEL). The MainSEL was piloted in the “Collaboration on Rare Diseases” (CORD_MI) project to calculate the set intersection cardinality among eight German university hospitals and medical research institutes.^32^ Furthermore, MainSEL is used in the OnkoFDZ^75^ project to link data from clinical cancer registries, health insurance providers, cancer research centers, and university hospitals of the German Cancer Consortium—where the unprecedented security and privacy guarantees of MainSEL made the linkage legally feasible in the first place. New possibilities in biomedical research opened up by SMPC-based algorithms represent an important and promising field of research.^76^^,^^77^^,^^78^

The integration of Mainzelliste’s API into several third-party tools underlines one key lesson learned: the technical integration is usually not as difficult as the administrative and legal challenges. The real-world usage of systems such as Mainzelliste must be meticulously described in DPCs, consent forms must be written and approved, and data-sharing processes must be defined and harmonized between institutions. If those medico-legal challenges are not taken seriously, the timely establishment of a pseudonymization and ID management infrastructure is in jeopardy.

Most importantly, Mainzelliste embodies “living open source,” going beyond merely providing the source code under a free/libre open-source license. During the last 10 years, an active community has formed around Mainzelliste that not only uses the software but also contributes to its development. While we act as a “steward” for Mainzelliste’s source code repository, collaborators across the medical informatics domain contribute new features to Mainzelliste to suit their specific project needs and for the open-source community to reuse. Contributions include Soundex and LSH-based blocking (see performance considerations), generation and management of audit trails, support for HL7 FHIR, support for German health insurance identifiers as a pseudonym type, and the MainSEL SMPC-based record linkage extension (see PPRL).

Ultimately, Mainzelliste’s community-based development and widespread use over the last decade show that even functionality critical for data protection can be developed “by researchers, for researchers” and is a testament to sustainable open-source academic software development.

## Resource availability

### Lead contact

Requests for further information and resources should be directed to the lead contact, Galina Tremper (g.tremper@dkfz.de). The Mainzelliste support team can be contacted via info@mainzelliste.de.

### Materials availability

This paper presents an open-source software that does not generate any shareable materials. For the source code, see data and code availability.

### Data and code availability

• The most recent source code for Mainzelliste and the Mainzelliste GUI can be found here:
  • Mainzelliste: https://bitbucket.org/medicalinformatics/mainzelliste/.
  • Mainzelliste GUI: https://github.com/medicalinformatics/mainzelliste-gui.
• An archived copy of the source code at the time of publication is available on Zenodo^32^ under Zenodo: https://doi.org/10.5281/zenodo.17375765.

## Acknowledgments

The authors would like to thank all users of Mainzelliste who contributed valuable feedback or even source code, extending its functionality. In particular, we would like to thank Meet Bhatt, Andreas Borg, Johannes Drepper, Martin Franke, Benjamin Gathmann, Manuel Grün, Kay Hamacher, Hauke Hund, Cornelius Knopp, Christian Koch, Matthias Lemmer, Daniel Menzel, Marlena Meyer, Torsten Panholzer, Marcel Parciak, Erhard Rahm, Jürgen Riegel, Florens Rohde, Ulrich Sax, Francesco Scalia, Josef Schepers, Phillipp Schoppmann, Franziska Schramm, Lens Schwanke, Ziad Sehili, Alexander Stahmann, Florian Stampe, Markus Suhr, Christian Syska, and Daniel Volk. We thank Marcus Buchwald, Johannes Haebe, Vincent Heuveline, Stefan Machmeier, Olaf Pichler, and Felix Schledorn of the University Computing Center of Heidelberg University and the Engineering Mathematics and Computing Lab for conducting an independent white-box penetration test of Mainzelliste. Mainzelliste would not have been possible without the work of Klaus Pommerening (1946–2023), who dedicated his professional life to pragmatic solutions for data protection in medical research. This work has been supported by the 10.13039/501100001659German Research Foundation (DFG) with grant number LA 3859/1-1, the 10.13039/501100012353German Cancer Consortium (DKTK), and the Hector II Foundation as part of the DKFZ-Hector Cancer Institute at University Medicine Mannheim and the Helmholtz Institute for Translational Oncology (HI-TRON) Mainz.

## Author contributions

Conceptualization and supervision, M.L.; programming, G.T., M.B.A., T.B., T.K., and M.L.; manuscript writing, G.T., T.B., M.B.A., M.L., and T.K. All authors thoroughly reviewed and edited the manuscript.

## Declaration of interests

The authors have nothing to declare.
